# Prediction of late adverse events in pelvic cancer patients receiving definitive radiotherapy using radiation-induced gamma-H2AX foci assay

**DOI:** 10.1093/jrr/rrad079

**Published:** 2023-10-15

**Authors:** Masanori Someya, Tomokazu Hasegawa, Asako J Nakamura, Takaaki Tsuchiya, Mio Kitagawa, Toshio Gocho, Sho Mafune, Yutaro Ikeuchi, Hiroshi Tauchi, Koh-ichi Sakata

**Affiliations:** Department of Radiology, School of Medicine, Sapporo Medical University, S1W16, Chuo-ku, Sapporo, Hokkaido 060-8543, Japan; Department of Radiology, School of Medicine, Sapporo Medical University, S1W16, Chuo-ku, Sapporo, Hokkaido 060-8543, Japan; Department of Biological Sciences, College of Sciences, Ibaraki University, Bunkyo 2-1-1, Mito, Ibaraki 310-8512, Japan; Department of Radiology, School of Medicine, Sapporo Medical University, S1W16, Chuo-ku, Sapporo, Hokkaido 060-8543, Japan; Department of Radiology, School of Medicine, Sapporo Medical University, S1W16, Chuo-ku, Sapporo, Hokkaido 060-8543, Japan; Department of Radiology, School of Medicine, Sapporo Medical University, S1W16, Chuo-ku, Sapporo, Hokkaido 060-8543, Japan; Department of Radiology, School of Medicine, Sapporo Medical University, S1W16, Chuo-ku, Sapporo, Hokkaido 060-8543, Japan; Department of Radiology, School of Medicine, Sapporo Medical University, S1W16, Chuo-ku, Sapporo, Hokkaido 060-8543, Japan; Department of Biological Sciences, Faculty of Sciences, Ibaraki University, Bunkyo 2-1-1, Mito, Ibaraki 310-8512, Japan; Department of Radiology, School of Medicine, Sapporo Medical University, S1W16, Chuo-ku, Sapporo, Hokkaido 060-8543, Japan

**Keywords:** gamma-H2AX focus, radiosensitivity, late toxicity, adverse event

## Abstract

Radiation can induce DNA double-stranded breaks, which are typically detected by the fluorescence of phosphorylated histone H2AX. In this study, we examined the usefulness of the dynamics of radiation-induced gamma-H2AX foci of peripheral blood lymphocytes (PBLs), as a marker of DNA repair ability, in predicting late adverse events from radiotherapy. A total of 46 patients with cervical, vaginal and anal canal cancers treated with radical radiotherapy between 2014 and 2019 were included in this analysis. Concurrent chemotherapy was administered in 36 cases (78.3%). Peripheral blood was obtained before treatment, and then irradiated *ex vivo* with 1 Gy X-ray. The ratio of radiation-induced gamma-H2AX foci in PBLs measured at 30 min and at 4 h was defined as the foci decay ratio (FDR). With a median follow-up of 54 months, 9 patients (19.6%) were observed to have late genitourinary or gastrointestinal (GU/GI) toxicity. The FDR ranged from 0.51 to 0.74 (median 0.59), with a significantly higher incidence of Grade 1 or higher late adverse events in the FDR ≥ 0.59 group. In multivariate analysis, FDR ≥ 0.59 and hypertension also emerged as significant factors associated with the development of late toxicities. Overall, our results suggest that measurement of radiation-induced gamma-H2AX foci in PBLs may predict the risk of late GU/GI toxicities from chemoradiotherapy, which can enable tailoring the radiation dose to minimize adverse effects.

## INTRODUCTION

After conventional external beam irradiation for pelvic malignancies, some patients develop moderate or severe late adverse events. These symptoms may be permanent and could have a significant impact on quality of life. Common adverse events include hematuria, gastrointestinal bleeding, small bowel obstruction [i.e. genitourinary or gastrointestinal (GU/GI) toxicities] and pelvic bone insufficiency fractures. Although many risk factors for late radiation toxicity have been reported [1–3], there is still no reliable method to accurately predict the individual risk of radiation toxicity. If accurate predictions can be established, it may be possible to personalize treatment and thus prevent the development of serious late adverse events. Specifically, patients with a higher risk of late adverse effects of radiotherapy could be offered a lower dose of radiation, whereas patients with a minor risk of toxicity could be treated with a higher dose of radiation.

Clinical risk factors associated with the incidence of adverse events include advanced age, previous abdominal surgery and comorbidities, such as diabetes or collagen disease [4, 5]. Genetic factors are also widely recognized to influence radiosensitivity, with growing evidence to suggest these associations [6].

DNA double-strand breaks (DSBs) are the most lethal form of damage caused by ionizing radiation. The kinetics of DSBs can be monitored by immunofluorescent detection of phosphorylated histone H2AX (gamma-H2AX) [7], which is one of the earliest markers of DNA DSBs in lymphocytes. The peak of focus formation occurs ~30 min after X ray irradiation, and within 2 h ~80% of DSBs are repaired and focus formation is diminished [8]. Several reports have demonstrated that the ratio of decay of these foci may be used to evaluate the DNA damage repair ability of an individual [9–11].

In this study, we examined the usefulness of assessing the DNA repair capacity according to the H2AX foci assay in predicting the risk for the development of late adverse events, and the factors associated with this risk, in patients with cervical, vaginal and anal canal cancers, who were treated with definitive chemoradiotherapy.

## MATERIALS AND METHODS

### Patients

This observational study was approved by the Institutional Review Board of our institution (No. 25-191). A total of 46 patients with cervical, vaginal and anal cancers were included in the study, including 45 cases of squamous cell carcinoma and one case of adenosquamous cell carcinoma. All patients were treated with radical radiotherapy or concurrent chemoradiotherapy (CCRT) between 2014 and 2019. The characteristics of the patients are summarized in Table 1.

**Table 1:** Patient and treatment characteristics

		*n* = 46
Age		42–92 (65)
BMI		16.0–34.6 (22.1)
Hypertension		16 (34.8%)
DM		4 (8.7%)
Smoking		15 (32.6%)
Abdominal surgery		7 (15.2%)
Cancer site	Cervical cancer	43 (93.5%)
	Vaginal cancer	1 (2.2%)
	Anal cancer	2 (4.3%)
Pathology	SCC	45 (97.8%)
	AC	1 (2.2%)
Stage (UICC 8th)		
II		11 (23.9%)
III		19 (41.3%)
IV		16 (34.8%)
Pelvic lymph node metastasis		34 (73.9%)
Paraaortic lymph node metastasis		10 (21.7%)
Pretreatment tumor diameter (cm)		2.5–9.5 (6.1)
EBRT dose (whole pelvis) + ICBT point A dose		
30–30.6 Gy/ 15–17 fr	24 Gy/ 4 fr	3 (2.2%)
39.6–40 Gy/ 20–22 fr	18 Gy/ 3 fr	27 (64.4%)
50–50.4 Gy/ 25–28 fr	12 Gy/ 2 fr	8 (15.6%)
55.6–61.7 Gy/ 31–34 fr (EBRT alone)		8 (17.4%)
Total dose (Gy)		55.6–96.7 (68.6)
Concurrent chemotherapy		36 (78.3%)
Weekly CDDP or NDP		34 (73.9%)
MMC + Cape or S-1		2 (4.3%)
Adjuvant chemotherapy		20 (43.5%)

Median values are shown in parenthesis. Abbreviations: BMI = body mass index, DM = diabetes mellitus, SCC = squamous cell carcinoma, AC = adenocarcinoma, EBRT = external beam irradiation, ICBT = intracavitary brachytherapy, NDP = nedaplatin, MMC = mitomycin C, Cape = capecitabine. Total dose is sum of EBRT and ICBT dose converted to equivalent dose to 2 Gy.

### Treatment

Patients with cervical and vaginal cancer received box field external beam radiotherapy (EBRT) using 3D treatment planning, with a pelvic dose of 30–50 Gy and 4–20 Gy with central shielding, followed by 12–24 Gy of 192-Ir-high-dose rate intracavitary brachytherapy (ICBT). The combined EBRT and ICBT doses for the high-risk clinical target volume were calculated using the equivalent dose in 2 Gy fractions (EQD2) calculation, which was converted into 2 Gy conventional fractionation. Eight patients were treated with EBRT alone due to a larger tumor size or poorer general condition. Among the 45 included patients, 34 patients received CCRT, which involved weekly administration of cisplatin or nedaplatin at a dose of 40 mg/m^2^. Patients with anal cancer were treated with 59.4 Gy radiation therapy delivered in 33 intensity-modulated fractions with concurrent administration of Mitomycin C and capecitabine. Twenty patients were given adjuvant chemotherapy at their gynecologist’s discretion, which involved 3–6 cycles of either paclitaxel and carboplatin therapy or nedaplatin and irinotecan therapy.

### Immunofluorescent staining for gamma-H2AX in peripheral blood lymphocytes

**Fig. 1:**
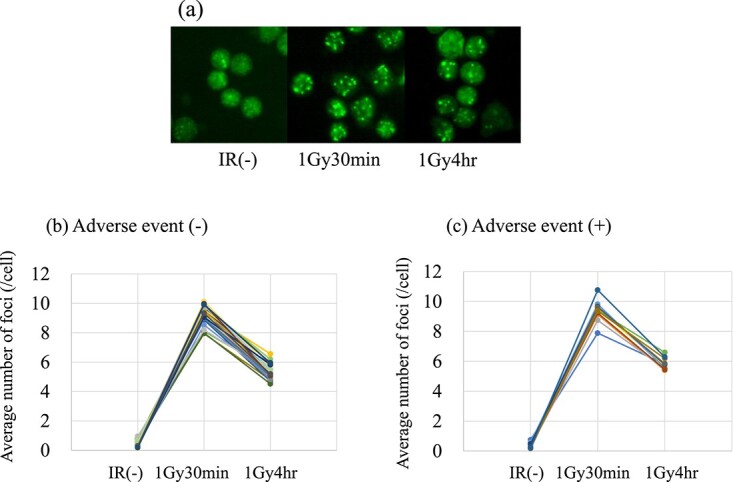
(a) Representative images of fluorescent immunostaining of radiation-induced gamma-H2AX foci. IR(−): non-irradiated; 1Gy30min: 30 min after 1 Gy of X-ray irradiation; 1Gy4hr: 4 h after 1 Gy of X-ray irradiation. (b) Time course of the average number of gamma-H2AX foci with no adverse event. (c) Time course of the average number of gamma-H2AX foci with late adverse events.

Before treatment, 10 ml of peripheral blood was collected in a heparinized tube from all patients. The blood was irradiated *ex vivo* with 1 Gy X-ray of 120 kV at 1.17 Gy/min. The interval between blood collection and *ex vivo* irradiation was within 30 min in most cases. The samples were incubated for 30 min and for 4 h after irradiation, respectively, and then peripheral blood lymphocytes (PBLs) were separated with Lymphoprep (Nycomed Pharma AS), centrifuged at 1500 rpm (300 × g) for 30 min at 4°C, and washed twice with phosphate-buffered saline. PBLs were then fixed in 2% paraformaldehyde for 20 min at room temperature. Fixed PBLs were spread on glass slides and subject to fluorescent immunostaining using anti-phosphorylated H2AX (Millipore, Cat. #05-636) as the primary antibody, followed by incubation with Alexa-488-conjugated anti-rabbit IgG (Molecular Probes) as the secondary antibody for the visualization of foci. Gamma-H2AX foci were observed with a fluorescent microscope under 10 × 100 oil immersion (Olympus). Representative images of Gamma-H2AX foci were shown in Fig. 1a. For quantification of foci, clear and easily distinguished dots of certain brightness were counted as positive foci. The number of gamma-H2AX foci was counted in at least 100 cells of each sample for each time point by visual inspection and the average number of foci per cell was calculated. The ratio of the number of foci detected after 30 min to that detected after 4 h of 1 Gy irradiation was calculated as the foci decay ratio (FDR). Figure 1b and c shows the time course of the foci count for each case with or without late events. Although there was a learning curve in the visual foci counts according to the experience of the researcher (data not shown), the FDR was almost constant (Supplementary Fig. S1); therefore, we considered the effect of learning to be negligible.

### Evaluation of adverse events

Acute and late adverse events were evaluated according to the National Cancer Institute Common Toxicity Criteria, version 4.0 (CTCAE 4.0). In regard to late adverse events, patients who were alive for at least 1 year after treatment were included in the evaluation. The median follow-up time was 54 months (range, 16–94 months). The cut-off date for this analysis was September 2022.

### Statistical analysis

Unpaired *t*-tests were used to compare the means of the number of foci in 30 min, 4 h and FDRs in Grades 0, 1 and 2 of the late GU/GI toxicities, respectively. The cumulative incidence of late adverse effects was estimated and the significance of differences in incidence was determined by Gray’s test. Cox proportional hazards analysis and logistic regression analysis were used to identify variables that influenced adverse events. The cut-off value was determined by receiver operating characteristic analysis. Correlations between blood cell counts and FDR were examined by Spearman’s rank correlation coefficient analysis. All statistical analyses were performed with BellCurve for Excel 2.00 (BellCurve, Tokyo, Japan).

## RESULTS

At the time of analysis, 8 of the 46 patients had died of the disease and 6 were alive with recurrent disease. Acute and late adverse events were shown in Supplementary Table S1 and Table 2. In regard to late adverse events, radiation proctitis was observed in seven patients, radiation cystitis in one patient and ureteral stricture in one patient (Table 2). D2cc of the rectum ranged from 40.0 to 86.9 Gy (median 62.4 Gy) and D2cc of the bladder ranged from 40.0 to 103.2 Gy (median 68.6 Gy).

**Table 2:** Late adverse events

	CTC-AE Grade			
	0	1	2	3
Radiation proctitis	39 (84.8%)	3 (6.5%)	3 (6.5%)	1 (2.2%)
Radiation cystitis	45 (97.8%)	1 (2.2%)	0	0
Ureteral stricture	45 (97.8%)	0	1 (2.2%)	0
Pelvic fracture	33 (71.7%)	12 (26.1%)	1 (2.2%)	0

### Time course of gamma-H2AX foci formation

**Fig. 2:**
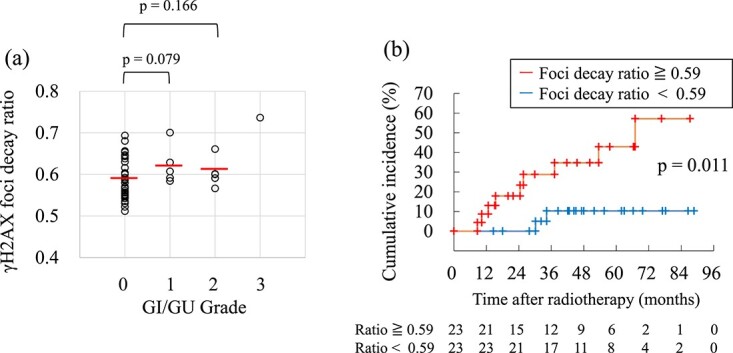
Associations of gamma-H2AX foci with late adverse events after radiotherapy. (a) Association between the gamma-H2AX FDR and grade of late GI/GU toxicity. Red horizontal bars indicate mean values. (b) Cumulative incidence curves of Grade 1 or higher late GU/GI toxicities stratified by the gamma-H2AX FDR (≥0.59 vs <0.59).

After 30 min and 4 h of 1 Gy irradiation, the average H2AX focus in PBLs was 9.3 foci/cell (range, 7.9–10.8) and 5.4 foci/cell (range, 4.0–6.6), respectively. The FDR ranged from 0.51 to 0.74 with a median of 0.59 (Fig. 2a). When the patients were divided into two groups with and without late adverse events, the mean values at 30 min were 9.3 foci/cell (range, 7.9–10.8) and 9.4 foci/cell (range, 7.9–10.1), respectively, and there was no statistically significant difference between the two groups (*P* = 0.690). In contrast, the mean values at 4 h were 5.8 foci/cell (range, 5.4–6.6) in the group with late adverse events and 5.4 foci/cell (range, 4.5–6.6) in the group without adverse events, respectively, showing a statistically significant difference between the two groups (*P* = 0.008).

### Predictive factors of late GU/GI toxicities

The relationship between late GU/GI toxicity and the FDR is shown in Fig. 2a. There was a trend toward higher mean FDR values for Grades 1–3 compared to Grade 0 toxicities; however, the difference was not statistically significant. The cut-off FDR value for predicting adverse events in receiver operating characteristic analysis was determined to be 0.59 (Supplementary Fig. S2), which was consistent with the median FDR for all cases. When the patients were divided into two groups by the cut-off value, the cumulative incidence of Grade 1 or higher adverse events was significantly higher in the high FDR group (FDR > 0.59) (Fig. 2b, *P* = 0.011).

In univariate analysis, FDR, age over 65 years, hypertension, clinical target volume dose of 70 Gy or more and maximal bladder dose of 90 Gy or more emerged as significant factors associated with late GU/GI toxicities; in multivariate analysis, only hypertension and FDR were independent predictive factors for late GU/GI toxicities, whereas maximal bladder and rectum dose were not significant factors (Table 3).

**Table 3:** Univariate and multivariate analyses for prediction of GI/GU toxicities

Parameter	Cumulative incidence function			Factors for adverse event
	HR	95% CI	*P* value	
Univariate analysis				
Age (<65 y vs ≧65 y)	2.717	0.824–8.962	0.101	≧65 y
BMI (<23 vs ≧23)	1.300	0.363–4.655	0.687	
Hypertension (No vs Yes)	3.468	0.948–12.69	0.060	Hypertension
DM (No vs Yes)	2.453	0.520–11.58	0.257	
Smoking (No vs Yes)	0.615	0.130–2.903	0.615	
Abdominal surgery (No vs Yes)	0.753	0.094–6.048	0.790	
Foci decay ratio (<0.59 vs ≧0.59)	5.791	1.248–26.98	0.025*	≧0.59
Concurrent Chemotherapy				
(No vs Yes)	0.404	0.117–1.393	0.151	
ICBT (No vs Yes)	2.152	0.275–16.87	0.466	
CTV dose (≧70 Gy vs < 70 Gy)	3.485	0.898–13.53	0.071	≧70 Gy
Dmax Rectum (<75Gy vs ≧75 Gy)	1.351	0.291–6.262	0.701	
Dmax Bladder (<90Gy vs ≧90 Gy)	3.826	0.803–18.22	0.092	≧90 Gy
Pelvic fracture (No vs Yes)	0.504	0.108–2.341	0.382	
Multivariate analysis				
Hypertension (No vs Yes)	5.186	1.311–20.51	0.019*	Hypertension
Foci decay ratio (<0.59 vs ≧0.59)	6.540	1.333–32.08	0.021*	≧0.59
Dmax Rectum (<75Gy vs ≧75 Gy)	1.140	0.238–5.467	0.870	
Dmax Bladder (<90Gy vs ≧90 Gy)	3.469	0.599–20.10	0.165	

Abbreviations: HR = hazard ratio, CI = confidence interval, BMI = body mass index, DM = diabetes mellitus, ICBT = intracavitary brachytherapy, CTV = clinical target volume, Dmax Rectum / Bladder = maximum dose to the rectum / the bladder. Asterisks indicate statistically significant values (*P* < 0.05).

### Predictive factors of acute toxicities

The relationship between minimum count of lymphocyte and neutrophil during treatment and FDR were shown in Supplementary Fig. 3a and b. There seemed to be a weak inverse correlation between the minimum lymphocyte and neutrophil counts and FDR, but no statistical significance was demonstrated (lymphocyte: *r* = −0.184, *P* = 0.222; neutrophil: *r* = −0.157, *P* = 0.298). The relationship between FDR and other acute toxicities was shown in Supplementary Figure 4a–f. However, there was no correlation between FDR and grade of toxicity. In a multivariate analysis using logistic regression, the only significant factor affecting Grade 4 of lymhocytopenia was concurrent chemotherapy, while FDR and irradiated dose to CTV were not significant (Supplementary Table S2).

## DISCUSSION

Our results show that hypertension and the FDR of gamma-H2AX foci, which may reflect individual radiosensitivity, are significant predictive factors for the risk of late GI/GU toxicity, such as radiation proctitis and cystitis following radiotherapy for cervical, vaginal and anal cancers.

Several studies have used PBLs to predict radiosensitivity, particularly those examining rectal bleeding after radiotherapy for prostate cancer and acute radiation dermatitis in postoperative radiotherapy for breast cancer [10–12]. Ooschot *et al*. [10] analyzed PBLs from 34 patients in the over-responder group who had Grade 3 urinary tract or gastrointestinal late adverse events and 27 patients in the non-responder group who had Grade 0 adverse events >2 years after radiotherapy for prostate cancer. The gamma-H2AX focus was counted at 0.5 and 24 h after 1 Gy of X-ray irradiation to calculate the FDR, which was found to be significantly lower in the over-responder group. Subsequently, Nuijens *et al*. [11] analyzed FDR in 179 cases, including DVH parameters and clinical factors, and reported that transurethral resection of prostate, the volume of the rectum irradiated with 70 Gy and FDR were independent predictors of Grade 2 or higher urinary or gastrointestinal tract adverse events.

Mumbarkar *et al*. [9] performed immunostaining of gamma-H2AX at 0.25, 3 and 6 h after 2 Gy of X-ray irradiation using PBLs from patients undergoing postoperative radiotherapy for breast cancer, and found a higher number of residual gamma-H2AX foci (i.e. lower DNA repair capacity) in patients with Grade 2 or 3 acute skin reaction to radiotherapy compared with the number of foci found in the no skin reaction group. These studies used varied *ex vivo* radiation doses to PBLs and timing of foci measurements, and they involved relatively homogeneous populations treated with radiotherapy alone. In contrast, >70% of the patients included in our study had advanced cancer of Stage 3 or higher who received concurrent chemotherapy. Nevertheless, our results are consistent with the previous observations, demonstrating a general association between lymphocyte radiosensitivity and the development of adverse events.

The main limitations of this study are the relatively small sample size, heterogeneous patient backgrounds, varying treatment regimens and lack of an adequate validation study. Another limitation is the method of preparing slides for the radiation-induced gamma H2AX assay, which is time-consuming and requires skill in visual counting. Therefore, we expect that these procedures will be simplified and automated as much as possible to obtain more accurate measurements.

In conclusion, our results suggest the measurement of radiation-induced gamma H2AX foci in PBLs may predict the risk of late GU/GI toxicities from chemoradiotherapy, which can enable tailoring the radiation dose to minimize adverse effects.

## CONFLICT OF INTEREST

The authors declare that they have no conflicts of interest.

## FUNDING

This study was supported by Japan Society for the Promotion of Science (JSPS) KAKENHI (grants 21K07648, 21K07680, 22K07671, 23K07161 and 23K14923).

## DATA AVAILABILITY

All data including in this manuscript is available upon request.

## Supplementary Material

Supplementary_figure_R2_rrad079

Supplementary_Table1_R2_rrad079

Supplementary_Table2_R2_rrad079
