# Supplementary material for: Prediction of late adverse events in pelvic cancer patients receiving definitive radiotherapy using radiation-induced gamma-H2AX foci assay
Source: J Radiat Res. 2023 Oct 15;64(6):948–53. doi: 10.1093/jrr/rrad079 (PMC10665300; doi:10.1093/jrr/rrad079)
Supplement: Supplementary_figure_R2_rrad079 [file supplementary_figure_r2_rrad079.pptx]

## Slide 1
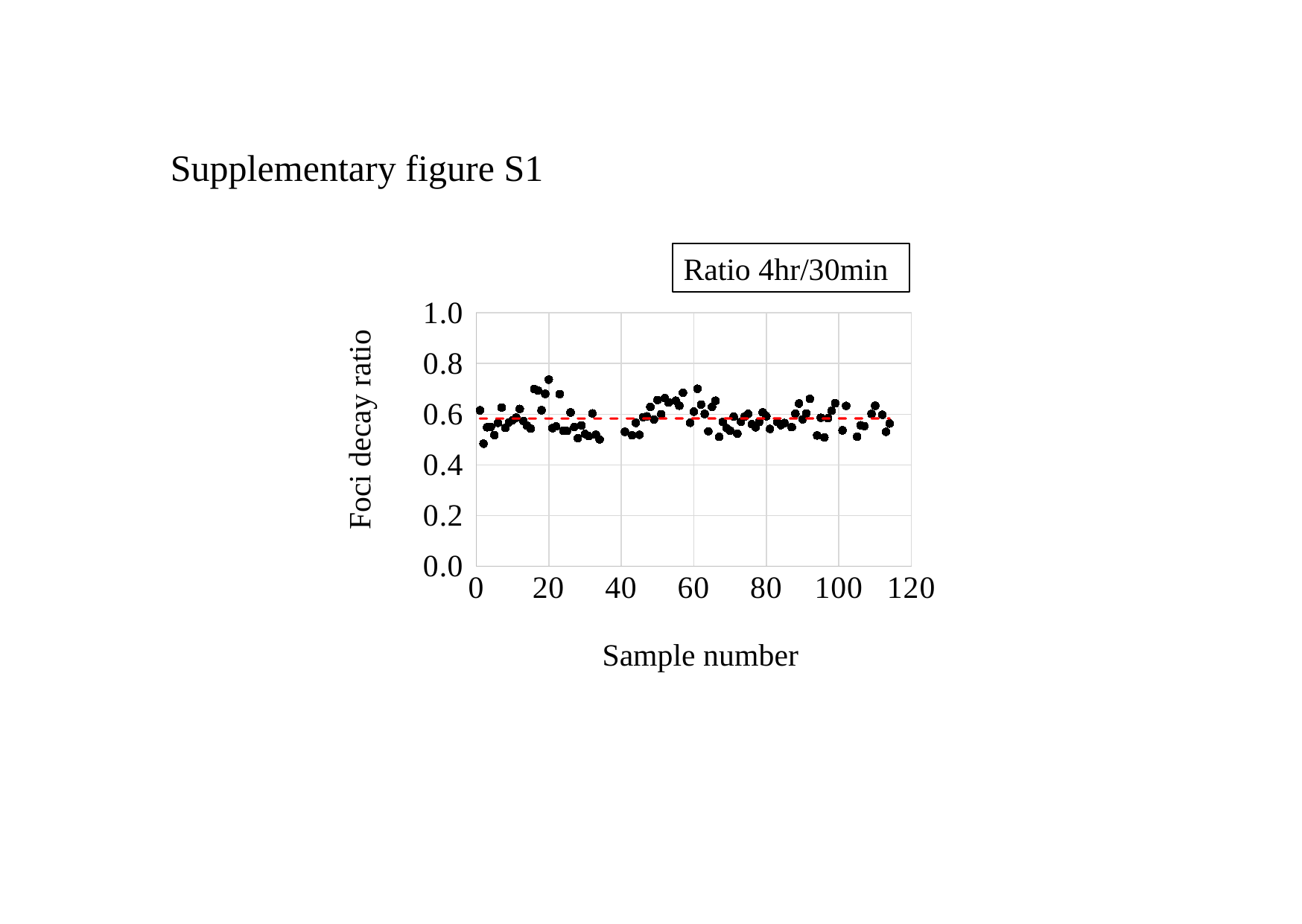

Supplementary figure S1
Ratio 4hr/30min
### Chart
| Category | |
|---|---|Foci decay ratio
Sample number

## Slide 2
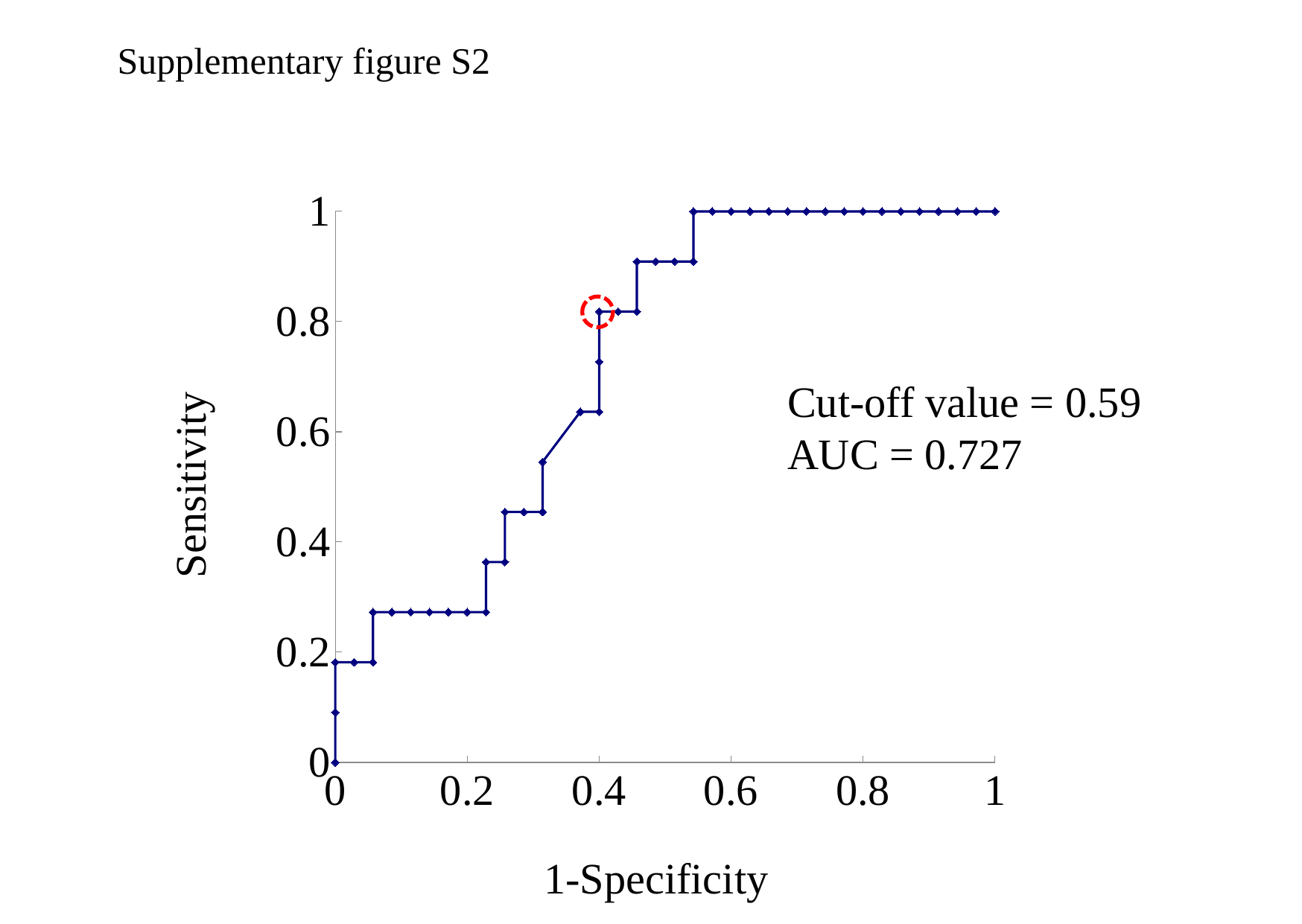

Supplementary figure S2
### Chart
| Category | repair rate |
|---|---|
Cut-off value = 0.59
AUC = 0.727
Sensitivity
1-Specificity

## Slide 3
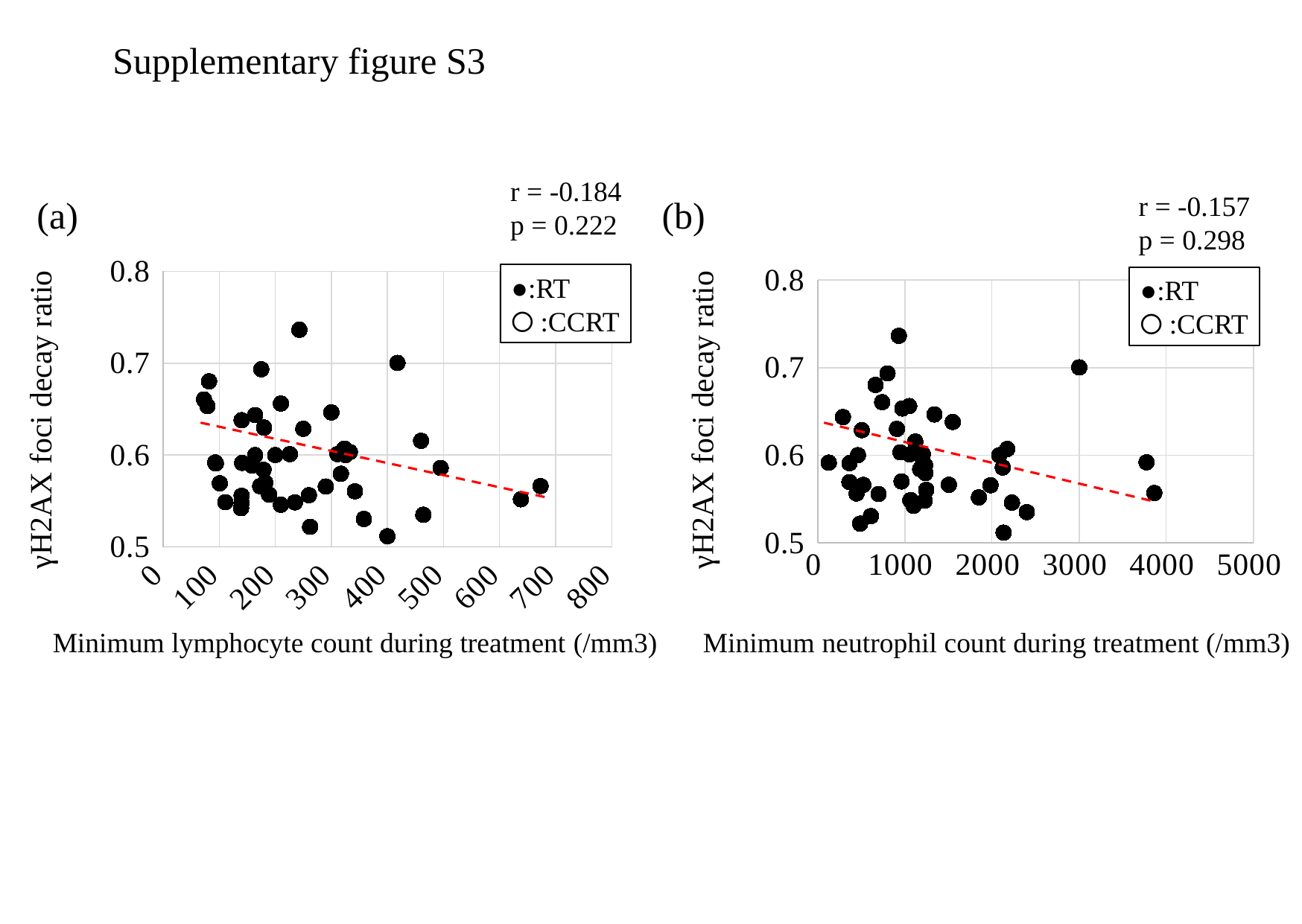

Supplementary figure S3
### Chart
| Category | | |
|---|---|---|r = -0.184
p = 0.222
r = -0.157
p = 0.298
(a)
(b)
### Chart
| Category | | |
|---|---|---|●:RT
〇:CCRT
●:RT
〇:CCRT
γH2AX foci decay ratio
γH2AX foci decay ratio
Minimum lymphocyte count during treatment (/mm3)
Minimum neutrophil count during treatment (/mm3)

## Slide 4
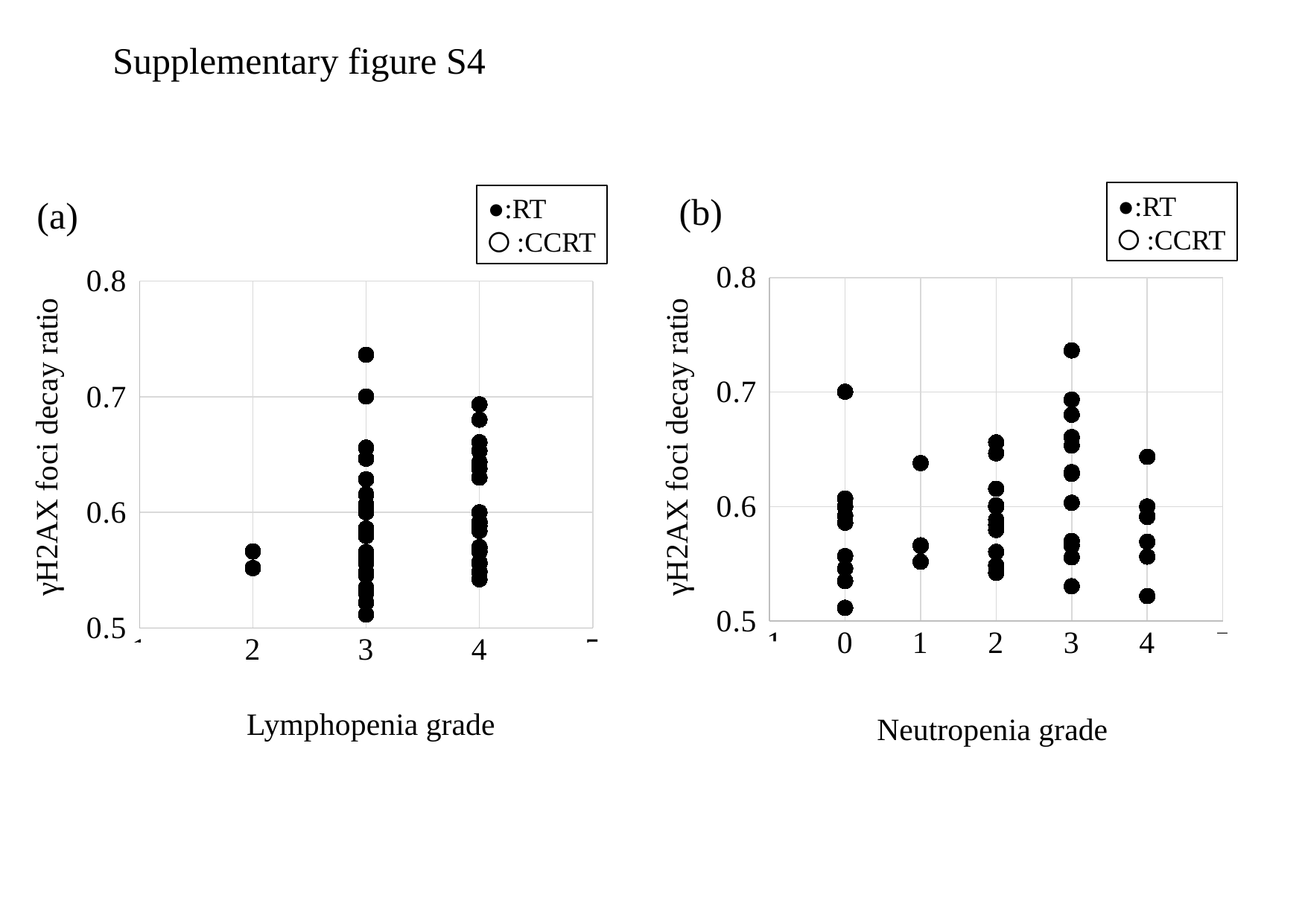

Supplementary figure S4
(b)
●:RT
〇:CCRT
●:RT
〇:CCRT
(a)
### Chart
| Category | | |
|---|---|---|
### Chart
| Category | | |
|---|---|---|γH2AX foci decay ratio
γH2AX foci decay ratio
Lymphopenia grade
Neutropenia grade

## Slide 5
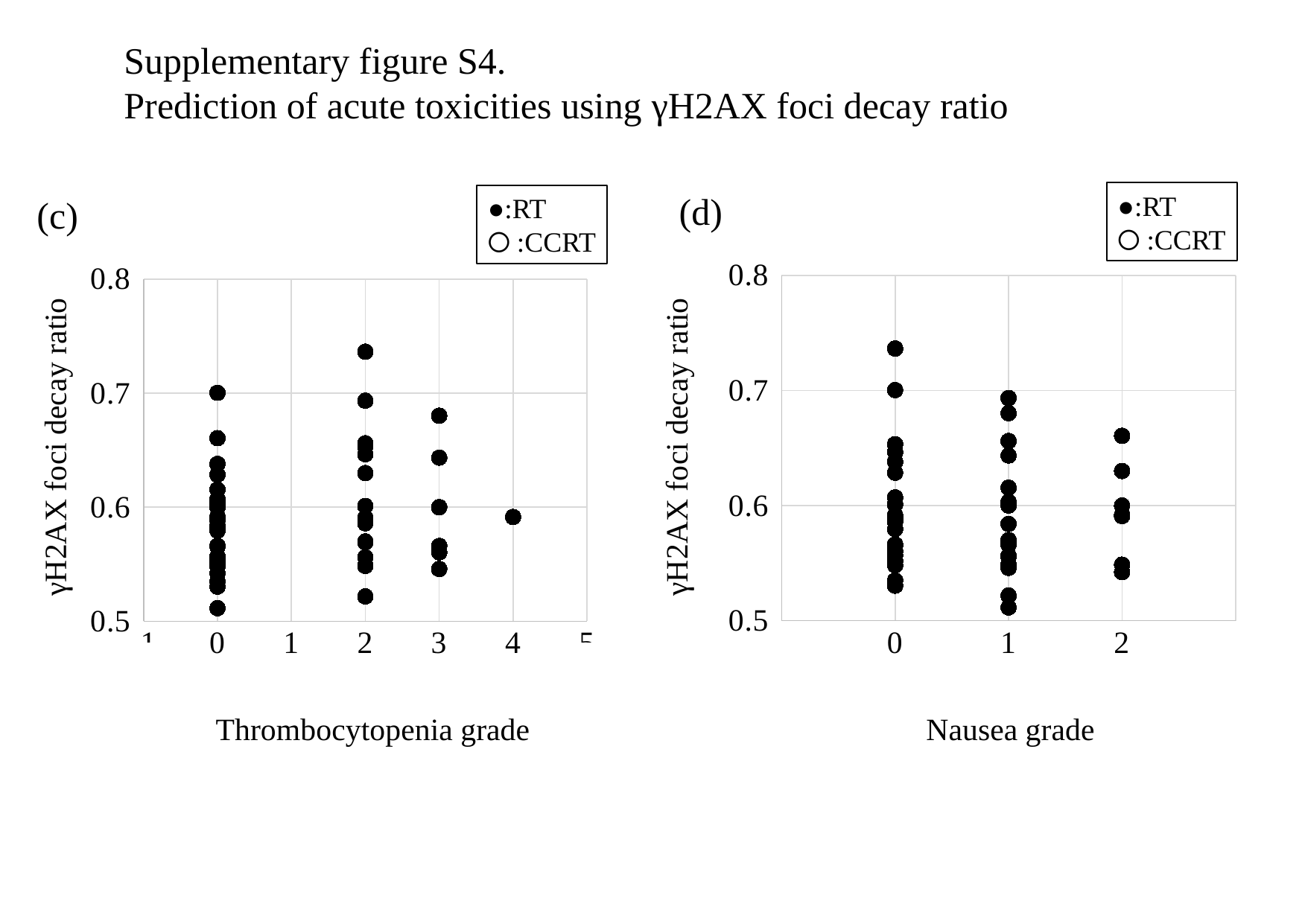

Supplementary figure S4.
Prediction of acute toxicities using γH2AX foci decay ratio
(d)
●:RT
〇:CCRT
●:RT
〇:CCRT
(c)
### Chart
| Category | | |
|---|---|---|
### Chart
| Category | | |
|---|---|---|γH2AX foci decay ratio
γH2AX foci decay ratio
Thrombocytopenia grade
Nausea grade

## Slide 6
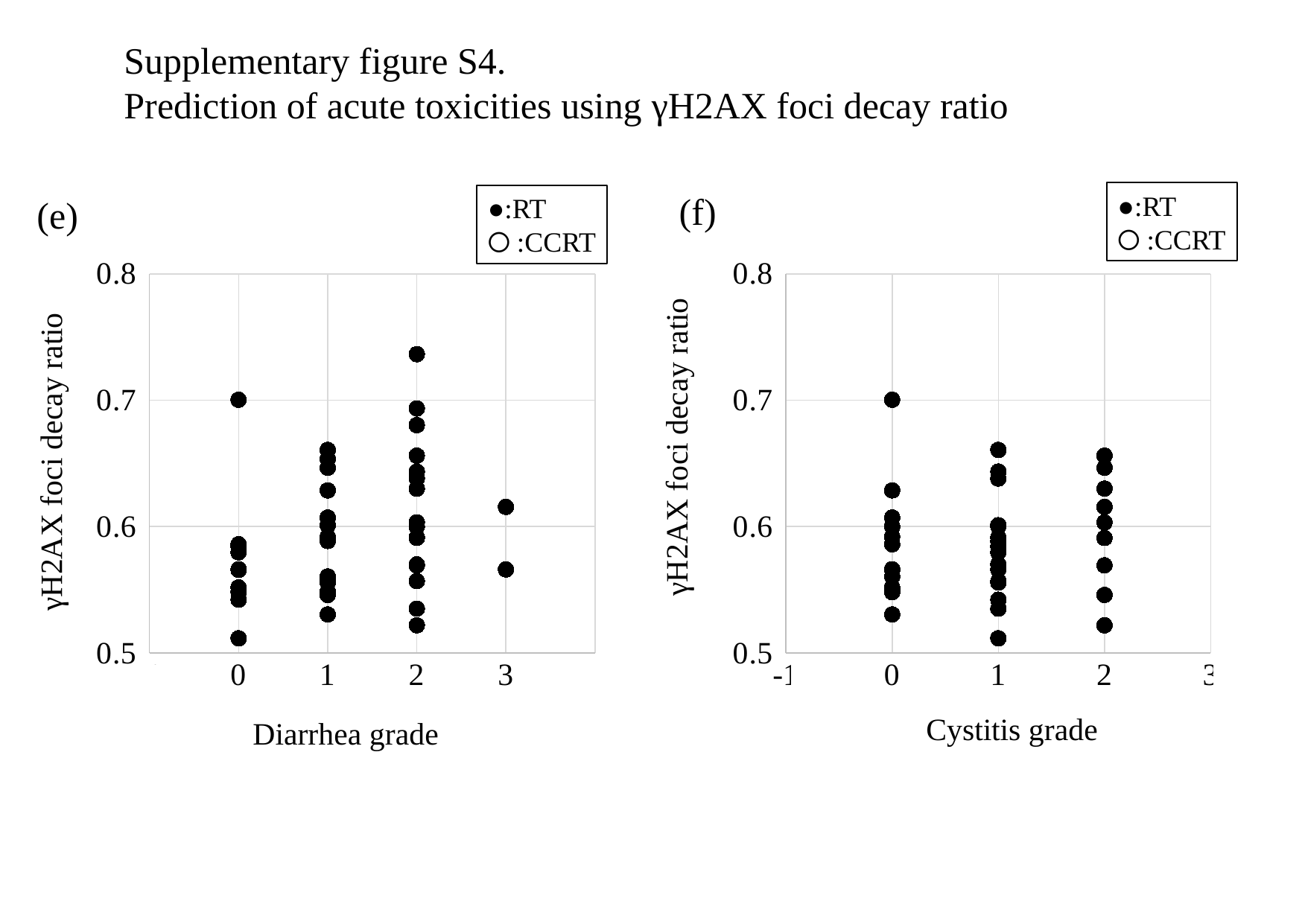

Supplementary figure S4.
Prediction of acute toxicities using γH2AX foci decay ratio
(f)
●:RT
〇:CCRT
●:RT
〇:CCRT
(e)
### Chart
| Category | | |
|---|---|---|
### Chart
| Category | | |
|---|---|---|γH2AX foci decay ratio
γH2AX foci decay ratio
Cystitis grade
Diarrhea grade
