# Supplementary material for: Prediction of late adverse events in pelvic cancer patients receiving definitive radiotherapy using radiation-induced gamma-H2AX foci assay
Source: J Radiat Res. 2023 Oct 15;64(6):948–53. doi: 10.1093/jrr/rrad079 (PMC10665300; doi:10.1093/jrr/rrad079)
Supplement: Supplementary_Table1_R2_rrad079 [file supplementary_table1_r2_rrad079.doc]

Supplementary Table 1. Acute adverse events

CTC-AE Grade

0 1 2 3 4

Anemia 2 (4.3%) 13 (28.3%) 12 (26.1%) 19 (41.3%) 0

Neutropenia 9 (19.6%) 4 (8.7%) 14 (30.4%) 12 (26.1%) 7 (15.2%)

Lymphopenia 0 0 2 (4.3%) 23 (50.0%) 21 (45.7%)

Thrombocytopenia 25 (54.3%) 0 14 (30.4%) 6 (13.0%) 1 (2.2%)

Nausea 20 (43.5%) 19 (41.3%) 7 (15.2%) 0 0

Diarrhea 10 (21.7%) 16 (34.9%) 18 (39.1%) 2 (4.3%) 0

Cystitis* 14 (30.4%) 17 (37.0%) 9 (19.6%) 0 0

*Six patients were not assessed due to percutaneous nephrostomy or ureteral stent.
