# Supplementary material for: Prediction of late adverse events in pelvic cancer patients receiving definitive radiotherapy using radiation-induced gamma-H2AX foci assay
Source: J Radiat Res. 2023 Oct 15;64(6):948–53. doi: 10.1093/jrr/rrad079 (PMC10665300; doi:10.1093/jrr/rrad079)
Supplement: Supplementary_Table2_R2_rrad079 [file supplementary_table2_r2_rrad079.doc]

Supplementary Table 2. Logistic analysis of predictors for ≥ Grade 4 lymphopenia

Factors for

Variables OR 95% CI p value lymphopenia

**Multivariate analysis**

Concurrent Chemotherapy

(No vs Yes) 2.596 1.222 – 8.961 0.011* Chemotherapy

CTV dose (≧70 Gy vs < 70 Gy) -0.111 -0.403 – 0.181 0.447

Foci decay ratio (<0.59 vs ≧0.59) -0.018 -0.314 – 0.278 0.902

Abbreviations: OR = Odds ratio, CI = Confidence interval. Asterisks mean statistically significant (p<0.05).
